# Highly Stable and Enhanced Performance of p–i–n Perovskite Solar Cells via Cuprous Oxide Hole-Transport Layers

**DOI:** 10.3390/nano13081363

**Published:** 2023-04-14

**Authors:** Tung-Han Chuang, Yin-Hung Chen, Shikha Sakalley, Wei-Chun Cheng, Choon Kit Chan, Chih-Ping Chen, Sheng-Chi Chen

**Affiliations:** 1 Institute of Materials Science and Engineering, National Taiwan University, Taipei 106, Taiwan; 2 Department of Mechanical Engineering, National Taiwan University of Science and Technology, Taipei 106, Taiwan; 3 Department of Materials Engineering and Center for Plasma and Thin Film Technologies, Ming Chi University of Technology, New Taipei City 243, Taiwan; 4 Mechanical Engineering Department, Faculty of Engineering and Quantity Surveying, INTI International University, Nilai 71800, Negeri Sembilan, Malaysia; 5 College of Engineering and Center for Green Technology, Chang Gung University, Taoyuan 333, Taiwan

**Keywords:** Cu_2_O films, solar cell, DCMS, HiPIMS, superimposed HiPIMS, hole-transport layer (HTL), power conversion efficiency (PCE)

## Abstract

Solar light is a renewable source of energy that can be used and transformed into electricity using clean energy technology. In this study, we used direct current magnetron sputtering (DCMS) to sputter p-type cuprous oxide (Cu_2_O) films with different oxygen flow rates (f_O2_) as hole-transport layers (HTLs) for perovskite solar cells (PSCs). The PSC device with the structure of ITO/Cu_2_O/perovskite/[6,6]-phenyl-C_61_-butyric acid methyl ester (PC_61_BM)/bathocuproine (BCP)/Ag showed a power conversion efficiency (PCE) of 7.91%. Subsequently, a high-power impulse magnetron sputtering (HiPIMS) Cu_2_O film was embedded and promoted the device performance to 10.29%. As HiPIMS has a high ionization rate, it can create higher density films with low surface roughness, which passivates surface/interface defects and reduces the leakage current of PSCs. We further applied the superimposed high-power impulse magnetron sputtering (superimposed HiPIMS) derived Cu_2_O as the HTL, and we observed PCEs of 15.20% under one sun (AM1.5G, 1000 Wm^−2^) and 25.09% under indoor illumination (TL-84, 1000 lux). In addition, this PSC device outperformed by demonstrating remarkable long-term stability via retaining 97.6% (dark, Ar) of its performance for over 2000 h.

## 1. Introduction

Solar energy is the most abundant and promising alternative to fossil energy. It can be collected and converted easily in the form of electricity for industrial and household usage via various technologies such as silicon-based solar cells (SSCs) [1], dye-sensitized solar cells (DSSCs) [2], organic photovoltaics (OPVs) [3,4], and perovskite solar cells (PSCs) [5,6]. Among these, PSCs have gained the most interest due to their impressive power conversion efficiency (PCE) [7,8,9,10]. The first PSC, introduced by Miyasaka in 2009, had a PCE of 3.8% [11], which now exceeds 25.7% [12] within quite a few years of development. Because of their easy fabrication in low temperatures, inexpensive solution process, and excellent performance attributed to outstanding optoelectronic properties [13,14,15,16,17,18,19,20,21,22,23,24,25], PSCs have attracted more attention. Perovskites typically have the chemical formula ABX_3_, where A is a monovalent cation (such as methyl ammonium [CH_3_NH_3_^+^ (MA)], NH = CHNH_3_^+^ (FA), or Cs^+^), B is either a Pb^2+^ or Sn^2+^ divalent metallic cation, and X is a halide anion (I^−^, Br^−^, or Cl^−^) [26,27].

The classifications of PSC device architecture based on arrangement of heterojunctions are n−i−p (conventional) and p−i−n (inverted). The selection of materials for hole-transport layers (HTLs) shows the impact on the performance and stability of PSCs. Commonly used hole-transport materials (HTMs) are 2,2′,7,7′-tetrakis-(N, N-di-4-methoxyphenylamino)-9,9′-spirobifluorene (Spiro-OMeTAD) [28], poly(3,4-ethylene dioxythiophene) (PEDOT:PSS) [29], poly(3-hexylthiophene) (P3HT) [30], and poly[bis(4-phenyl) (2,4,6-trimethylphenyl)-amine] (PTAA) [31]. These organic materials become highly unstable when exposed to air (moisture) for a long time. Thereby, the replacement of organic HTMs with inorganic HTMs is being considered for comparatively higher stability. For instance, NiO_x_ has been used as an efficient HTL possessing an outstanding performance with excellent air stability [19,32,33]. Cuprous oxide (Cu_2_O) offers a band gap in the range of 2.2–2.8 eV with an enormous hole mobility of (100 cm^2^V^−1^s^−1^) [34,35,36] and the features of nontoxicity, abundance on the earth’s crust, and low manufacturing cost, which make it a candidate for HTLs of PSCs [37]. Relentless research on Cu_2_O-based, transparent thin-film PSCs has achieved significant theoretical energy conversion efficiency [38,39]. There are various chemical/solution and physical deposition methods to prepare Cu_2_O thin films, such as sol-gel [40], thermal oxidation [34], chemical vapor deposition [41], RF/DC sputtering [42,43], electrodeposition [44], pulsed laser deposition [45], and high-power impulse magnetron sputtering (HiPIMS) [46]. Even though Cu_2_O thin films have outstanding properties, it is still challenging to deposit uniform, dense, smooth, and pinhole-free layers for PSC application.

Various sputtering methods have been considered, such as direct current magnetron sputtering (DCMS) (i.e, a plasma process used for film deposition). The HiPIMS technique offers a high ionization rate of sputtered target atoms and high plasma density, which is advantageous for metal-oxide thin-film deposition [47]. Unfortunately, HiPIMS has the drawback of a low deposition rate, which limits its application. Here, middle-frequency (MF) pulses during the off-time of HiPIMS, also known as superimposed HiPIMS (HiPIMS+MF), step in to overcome the deposition rate of the HiPIMS process without sacrificing the target ionization rate [48]. In this work, we demonstrated Cu_2_O as the HTLs of PSCs from different sputtering modes (DCMS, HiPIMS, and superimposed HiPIMS). We analyzed the structural, morphological, and optoelectronic properties of Cu_2_O derived PSCs. The DC, HiPIMS, and superimposed HiPIMS derived devices showed performances of 7.12 ± 0.64, 9.42 ± 0.92%, and 13.04 ± 1.61%, respectively. To the best of our knowledge, the PCE of the superimposed HiPIMS derived PSCs is among the best reported for CH_3_NH_3_PbI_3_ (MAPbI_3_) based devices. Furthermore, the respective device showed remarkable stability by retaining 97.6% of its performance (i.e., storage in the dark [Ar] for over 2000 h). Hence, this study explains the effect of depositing TCO films using different sputtering modes on the PCEs and stability of PSC devices.

## 2. Materials and Methods

Cu_2_O films (10 nm) were deposited by DCMS, HiPIMS [49], and superimposed HiPIMS [48] techniques, subsequently, on an ITO substrate. Before spin-coating the perovskite precursor solution, we used plasma to modify the Cu_2_O surface for 30 s and coat the precursor solution smoothly. Details of the device fabrication and characterization of the materials are shown in the Supplementary Materials.

## 3. Results

**Figure 1:**
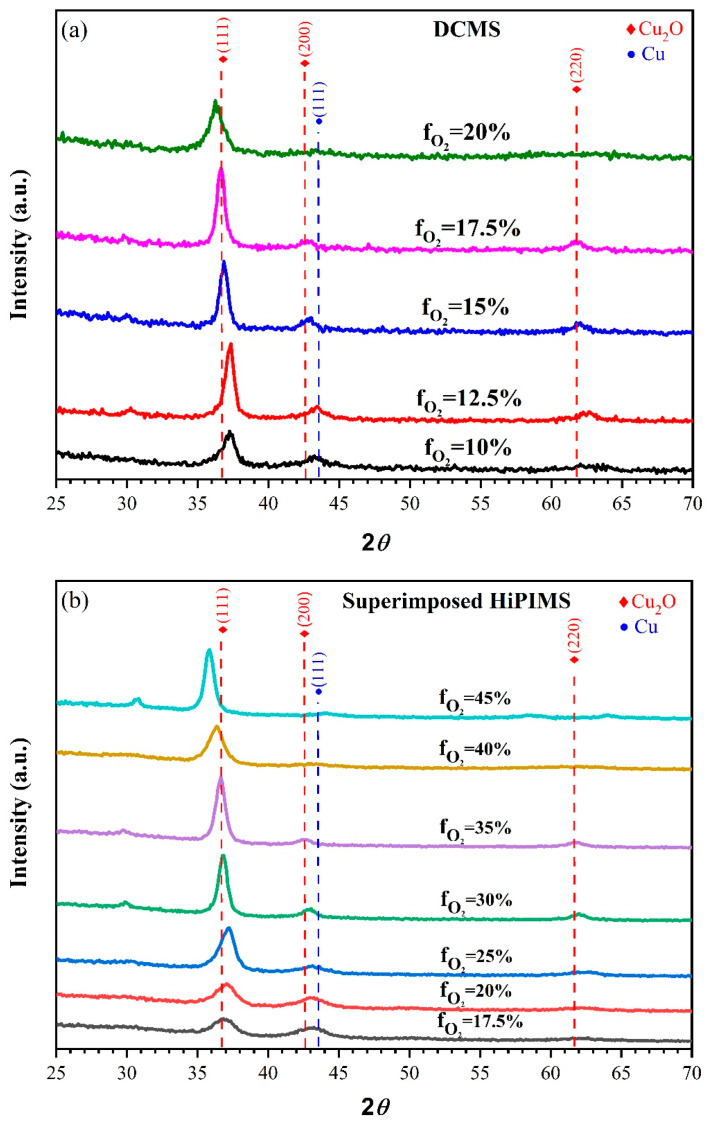
X-ray diffraction spectra of Cu_2_O films deposited by (**a**) DCMS and (**b**) superimposed HiPIMS at different oxygen flow rates.

We fabricated the PSCs with a p–i–n structure of Glass/ITO/Cu_2_O/MAPbI_3_/PC_61_BM/BCP/Ag. Figure 1 displays the X-ray diffraction patterns of Cu_2_O from DCMS and superimposed HiPIMS at different oxygen flow rates (f_O2_ = O_2_/(O_2_ + Ar) × 100%, Ar: Argon O_2_: Oxygen). For the DCMS (Figure 1a), the increase in oxygen flow rate from f_O2_ = 10% started to form the Cu_2_O phase and became pure Cu_2_O phase at f_O2_ = 17.5%. Beyond this, it started to form a mixed Cu_2_O phase. For the normal HiPIMS sample, we observed the Cu_2_O phase at f_O2_ = 20%, which is consistent with our previously published study [49]. For the XRD of the superimposed HiPIMS process (Figure 1b), the increase in oxygen flow rate from f_O2_ = 17.5% started to form the Cu_2_O phase and became pure Cu_2_O phase at f_O2_ = 30%, and later, with a further increase in f_O2_, it became a mixed-Cu_2_O phase. Accordingly, we obtained the optimal parameters for the Cu_2_O phase and used them as the HTLs of PSCs for further study.

**Figure 2:**
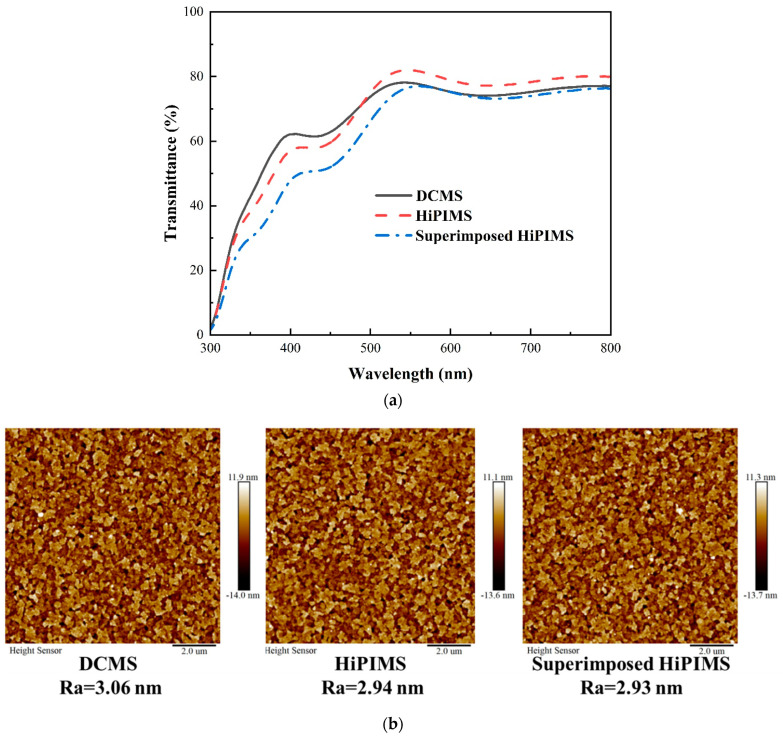
(**a**) UV-Vis spectra and (**b**) AFM topography images of Cu_2_O films deposited using various processes.

We determined the UV-Vis transmittance of these Cu_2_O-deposited ITO samples using air as a background (Figure 2a). The transmittance of the HiPIMS sample was slightly more than 80%, while in contrast, that of the DCMS and superimposed HiPIMS samples were each a bit less than 80% (<3%), in a wavelength range (500-900 nm). Due to the high ionization rate and high-energy plasma of the HiPIMS process, it offers dense and highly crystalline Cu_2_O films. Therefore, we observed higher transmittance of respective Cu_2_O in comparison with the DCMS sample (i.e., the structure was less dense). Although the superimposed HiPIMS process has the same advantages as the HiPIMS process, the MF power applied during the off-time bombarded more copper that became deposited as film, possibly contributing to the decrease in transmittance.

The values of Ra (the arithmetic average of the roughness profile) of the samples were determined by tapping-mode atomic force microscopy (AFM) to understand the surface morphology of ITO/Cu_2_O affecting the growth of perovskite; Figure 2b represents surface roughness through AFM images of the samples. We obtained values of Ra for the DCMS (3.06 nm), HiPIMS (2.94 nm), and superimposed HiPIMS (2.93 nm) samples. We speculated that HiPIMS would result in higher density and higher energy plasma than DCMS during deposition, which would result in a smoother surface. The superimposed HiPIMS sample had the same characteristics as the HiPIMS sample, leading to similar Ra values.

**Figure 3:**
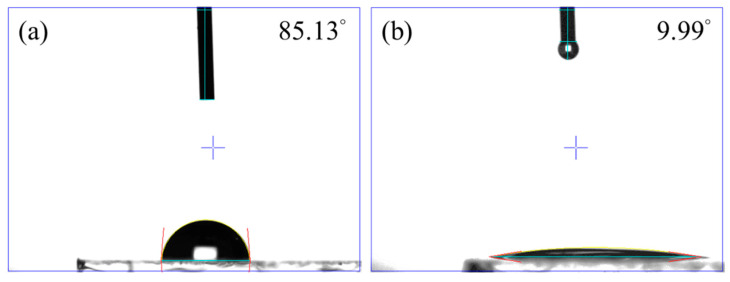
Cu_2_O film prepared by HiPIMS (**a**) before plasma cleaning and (**b**) after plasma cleaning of the contact angle.

Figure 3 shows the contact angles of the ITO/Cu_2_O surfaces when we dropped the perovskite precursor solution on top. As indicated in Figure 3a, the perovskite precursor formed a sphere on the HiPIMS Cu_2_O samples (i.e., a high angle of 85.13°), spreading poorly on the Cu_2_O surface and causing failure growth of the perovskite layer. The DCMS and superimposed HiPIMS Cu_2_O samples also had high angles, of 89.95° and 69.31°, respectively, as shown in Figure S1, which also led to poor spread of the perovskite precursor. To resolve this issue, we applied a plasma treatment to induce an ultraviolet effect that modified the surface of the Cu_2_O films. As indicated in Figure 3b, the Cu_2_O film became hydrophilic with a low angle of 9.99°, leading to the preferred growth of the perovskite layer.

**Figure 4:**
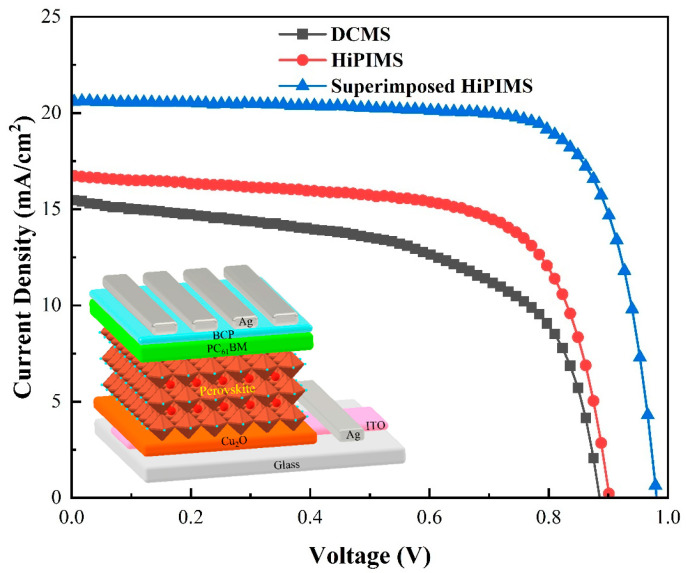
J–V curves of PSCs and device structure.

Table 1 and Figure 4 summarize the device performances for various Cu_2_O films. Table 1 displays the standard deviations of the PSCs calculated from a minimum of three samples deposited using the same technique. The perovskite deposition was from a solution, which may cause slight variations during each deposition. Due to the variability in optoelectronic and surface properties caused by the deposition process of HTLs, the PSCs produced using various conditions of HTLs exhibited different device performances. DCMS-derived devices provided a PCE of 7.12 ± 0.64%, with a short-circuit current density (J_sc_) of 13.74 ± 0.97 mAcm^−2^, a value of open-circuit voltage (V_oc_) of 0.89 ± 0.02 V, and a fill factor (FF) of 58.1 ± 3.93%. The HiPIMS-based devices showed slightly higher efficiency, with a PCE of 9.42 ± 0.92%, a value of J_sc_ of 17.36 ± 0.94 mAcm^−2^, a value of V_oc_ of 0.90 ± 0.00 V, and an FF of 61.1 ± 6.95%. The superimposed HiPIMS devices showed the highest performance, with a PCE of 13.04 ± 1.61%, a value of J_sc_ of 19.27 ± 1.20 mAcm^−2^, a value of V_oc_ of 0.98 ± 0.00 V, and an FF of 69.5 ± 4.98%. The improvement in the PCE of the overlaid HiPIMS PSCs was brought on by substantial increases in the J_sc_, V_oc_, and FFs. From the device’s J-V curves (Figure 4), we computed the series (R_s_) and shunt (R_sh_) resistances. The resistances of the electrodes, the interfacial resistance, the hole/electron transporting layers, and the perovskite all contributed to the value of R_s_. The highest value of R_s_ was for the DCMS device (9.47 Ω cm^2^), and the lowest value of R_s_ was for the superimposed HiPIMS device (1.42 Ω cm^2^). Further, we performed a four-point probe experiment to understand the electrical properties of Cu_2_O. This analysis showed the highest value of electrical conductivity (σ) for the superimposed HiPIMS Cu_2_O thin film (3.33 S cm^−1^) and the lowest for the DCMS Cu_2_O thin film (0.11 S cm^−1^). In addition, through XRD analysis (Figure S2), the crystallinity of the Cu_2_O improved from the DCMS to superimposed HiPIMS deposition techniques. According to previous studies, an increase in film’s crystallinity helps to increase the charge-carrier mobility (μ) [50]. In addition, varying the exposure times of the sputtering and plasma treatments impacted the optoelectronic properties of the thin films. In an attempt to reduce the exposure time during sputtering, we observed that the quality of the Cu_2_O film deteriorated significantly, potentially due to the film becoming discontinuous. Conversely, increasing the exposure time led to a well-formed Cu_2_O film. However, it resulted in unsatisfactory formation of the perovskite layer, and we are currently investigating the underlying reasons for this. As for the plasma treatment, we have not yet examined the impact of different exposure times on the optoelectronic properties of Cu_2_O film, as our primary goal was to improve its wettability. However, we speculate that excessively long exposure times during plasma treatment may damage Cu_2_O film and decrease its photoelectric properties. Lower values of R_s_ appear to be associated with higher σ and μ of Cu_2_O thin films and increased J_sc_ values of the device. We speculate that the increase in J_sc_ could have been due to a reduction in the carrier recombination rate at the p-i interface. This explanation is supported by other studies reporting that increasing hole conductivity can lead to higher J_sc_ [51]. We acknowledge that we do not have the necessary facilities to perform spectral-response characterization experiments, but our findings align with the existing literature. Overall, the use of superimposed HiPIMS Cu_2_O as HTLs resulted in higher FF, J_sc_, and PCE values compared with the devices based on DCMS and HiPIMS. As indicated in Figure 4, 15.20% was the best performance of the superimposed HiPIMS devices. Table 2 compares Cu_2_O films as the HTLs of PSCs prepared by different processes; V_oc_, J_sc_, FF, and PCE are the best performance values of the device. The Cu_2_O films were prepared by various methods such as spin coating [52,53], successive ionic layer adsorption and reaction (SILAR) [54], sputtering [55,56], the thermal oxidation method [57,58], electrodeposition [59,60], chemical vapor deposition (CVD) [61], etc. Although it is known from simulation results that, theoretically, the PCE of Cu_2_O-based PSCs can be as high as 25.2% [62], it was found that the actual device PCE was mostly in the range of 6 to 13%. Based on the above method, we observed that the improvement in PCE was due to the low series and high shunt resistance of the respective devices. The high conductivity of the Cu_2_O thin film resulted in low recombination loss and smaller bulk resistance of the device. These factors contributed to a reduction in the energy loss within the device and increased the efficiency of charge extraction and transfer. This phenomenon is consistent with findings in previous reports [54,60].

In this study, we achieved a competitive result of 15.21% via embedding Cu_2_O thin films using superimposed HiPIMS. The MF power-supply sputtering in the superimposed HiPIMS system allowed us to retain a high ionization rate as the advantage of the HiPIMS. Compared with DCMS processes, HiPIMS and superimposed HiPIMS can bombard sputtering ions with higher energy, which helps to form the preferred stoichiometry of Cu_2_O films, resulting in a denser coating with lower surface roughness. It helped to reduce the defects in the film and led to a smoother interface, resulting in a decrease in the leakage current and an improvement in the PCE. Our EPMA analysis (Table S1) showed that the Cu_2_O chemical composition ratio as Cu/O was 1.9, which is close to the form of perfect stoichiometric Cu_2_O.

**Figure 5:**
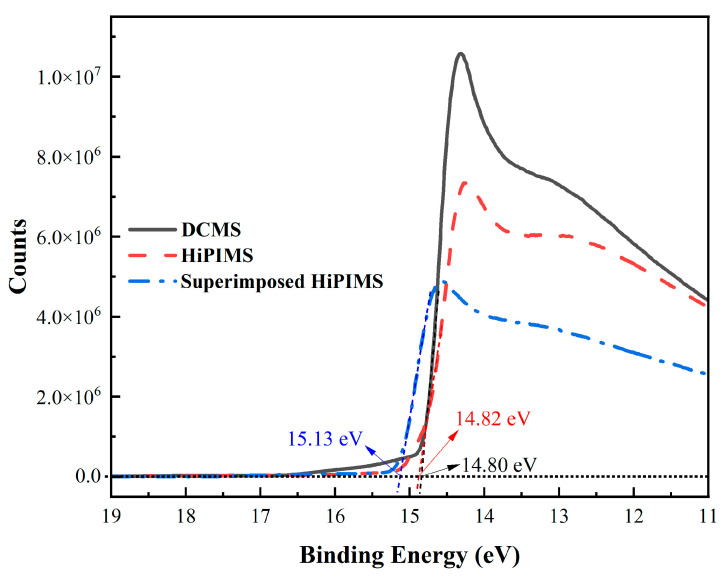
UPS spectra of ITO/Cu_2_O films (DCMS, HiPIMS, and superimposed HiPIMS).

In addition to evaluating the surface morphologies of the Cu_2_O films, we also evaluated the energy level alignment at the Cu_2_O/p-i interface using an incident light energy of 21.2 eV (He(I) emission) via ultraviolet photoelectron spectroscopy (UPS). Figure 5 presents the E_Cut-off_ regions of the DCMS, HiPIMS, and superimposed HiPIMS Cu_2_O samples (WF = 21.2 − E_Cut-off_) [63,64]. The DCMS Cu_2_O film had a work function (WF) of –6.40 eV, while the WF of the HiPIMS Cu_2_O film was –6.38 eV, which was 0.02 eV lesser than that of the DCMS Cu_2_O film. The WF of the superimposed HiPIMS sample was –6.07 eV (the negative work function means how much energy is required to be added to the bound electron by the photon it absorbs). Changes in the WF at the p-i interface can significantly impact the energy alignment of Cu_2_O with perovskite, hole transport, and effectiveness of charge extraction as well as transfer. Therefore, a shift in the energy levels of the Cu_2_O may have contributed to the improved performance of the Cu_2_O devices. We believe that the significantly different composition ratio (Table S1) and phase structure (Figure S2) of the superimposed HiPIMS Cu_2_O film may have affected the WF, resulting in the improved device performance.

**Figure 6:**
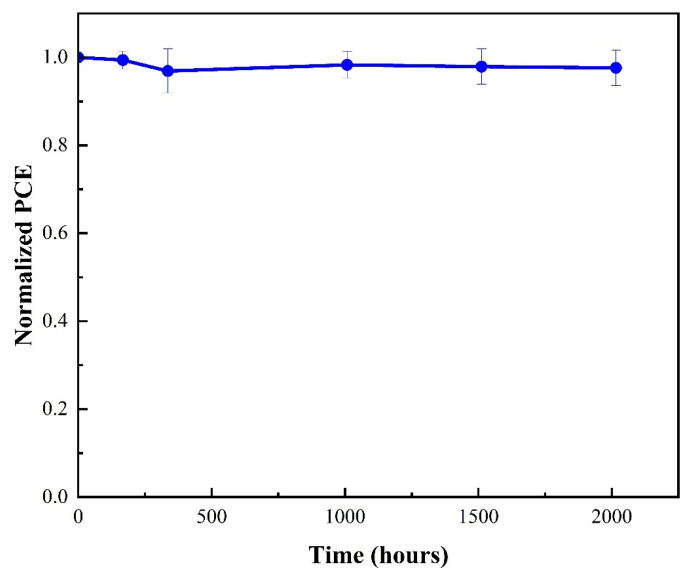
Stability data of unsealed devices from superimposed HiPIMS under the argon glove box.

We examined the shelf lives of the unencapsulated devices under an argon glove box in the dark to assess the stability of the superimposed HiPIMS Cu_2_O sample for PSC applications (we measured the PCE results at least three times to calculate the error bars). The devices initially offered the best PCE of 15.20%, as shown in Figure 6. After testing for 168 h, the devices retained 99.4% of their initial performance; after testing over 1000 h, the devices retained 98.3% of their initial performance; and after testing over 2000 h, the devices retained 97.6% of their initial performance. Thus, our PSC device suggested high stability. Finally, we determined the performance of the superimposed HiPIMS device under dim light (a TL-84 fluorescent lamp with an illumination of 1000 lux). Table 3 shows the performance of the respective device. The device showed a PCE of 25.09%. Based on these results, we demonstrated that Cu_2_O film has great potential as HTLs of PSCs under one sun and indoor illumination.

## 4. Conclusions

In this study, we prepared Cu_2_O films via DCMS, HiPIMS, and superimposed HiPIMS and used them as HTLs for PSC applications. In the comparison of different process technologies for Cu_2_O samples, superimposed HiPIMS enhanced the WF (measured by UPS), surface roughness (measured by AFM), and optoelectronic properties of the Cu_2_O films and, in turn, increased carrier extraction and transport at the perovskite–Cu_2_O interface. Because the optimized Cu_2_O film was obtained from superimposed HiPIMS technology, we observed an increase in the PCE of the PSCs from 7.91 (DCMS) to 15.20%. The extended shelf life of the unencapsulated devices in the glove box attested to their excellent stability and support Cu_2_O as a contender for p-type transporting layers in PSC applications.

## Figures and Tables

**Table 1:** Different process technologies for solar cell devices.

Sample	J_sc_ (mA/cm^2^)	V_oc_ (V)	FF (%)	PCE (%)	Best PCE (%)	R_s_^a^ (Ω cm^2^)	R_sh_^a^ (Ω cm^2^)	σ (S cm^−1^)
DCMS	13.74 ± 0.97	0.89 ± 0.02	58.1 ± 3.93	7.12 ± 0.64	7.91	9.47	111.82	0.11
HiPIMS	17.36 ± 0.94	0.90 ± 0.00	61.1 ± 6.95	9.42 ± 0.92	10.29	3.09	1081.18	0.40
Superimposed HiPIMS	19.27 ± 1.20	0.98 ± 0.00	69.5 ± 4.98	13.04 ± 1.61	15.20	1.42	1080.19	3.33

**Table 2:** Performances of Cu_2_O HTLs in PSCs prepared by different process technologies in the literature.

Process Technology	J_sc_ (mA/cm^2^)	V_oc_ (V)	FF (%)	PCE (%)	Year/Ref.
Spin Coating	16.52	1.07	75.5	13.35	2015/[52]
SILAR	16.52	0.89	56.0	8.23	2016/[54]
Rotating Sputtering	15.80	0.96	59.0	8.93	2016/[55]
Cu Thermal Oxidation	17.5	0.95	66.2	11.03	2016/[57]
Cu Thermal Oxidation	14.40	0.96	58.6	8.10	2016/[58]
Electrodeposition	18.03	0.88	61.0	9.64	2016/[59]
RF Sputtering	14.94	0.93	67.5	9.37	2018/[56]
CVD	17.50	0.98	48.0	8.26	2018/[61]
Electrodeposition	19.03	0.97	73.0	13.48	2019/[60]
Spin Coating	12.87	0.71	69.0	6.26	2021/[53]
Hydrothermal Technique	12.58	0.76	63.0	6.02	2022/[37]
Superimposed HiPIMS	20.34	0.98	76.5	15.20	This work

**Table 3:** Superimposed HiPIMS device with dim light.

Sample	J_sc_ (μA/cm^2^)	V_oc_ (V)	FF (%)	PCE (%)
Superimposed HiPIMS	189.51	0.62	66.4	25.09

## Supplementary Materials

The following supporting information can be downloaded at: https://www.mdpi.com/article/10.3390/nano13081363/s1, Experimental Details, Figure S1: Cu_2_O film prepared by (a) DCMS, (b) superimposed HiPIMS of contact angle.; Figure S2: X-ray diffraction spectra of different process technologies.; Table S1: Chemical composition of different process technologies. References [19,65,66] are cited in the supplementary materials.
