# Supplementary material for: Highly Stable and Enhanced Performance of p–i–n Perovskite Solar Cells via Cuprous Oxide Hole-Transport Layers
Source: Nanomaterials (Basel). 2023 Apr 14;13(8):1363. doi: 10.3390/nano13081363 (PMC10143474; doi:10.3390/nano13081363)
Supplement: Supplementary file 1 [file nanomaterials-13-01363-s001.zip › nanomaterials-2296002-supplementary.pdf]

Supplementary Material

Highly Stable and Enhanced Performance of p–i–n Perovskite Solar Cells via Cuprous Oxide Hole-Transport Layers

Tung-Han Chuang [1], Yin-Hung Chen [1], Shikha Sakalley [2,3], Wei-Chun Cheng [2], Choon Kit Chan [4], Chih-Ping Chen [3,*] and Sheng-Chi Chen [3,5,*]

[1] Institute of Materials Science and Engineering, National Taiwan University, Taipei 106, Taiwan
[2] Department of Mechanical Engineering, National Taiwan University of Science and Technology, Taipei 106, Taiwan
[3] Department of Materials Engineering and Center for Plasma and Thin Film Technologies, Ming Chi University of Technology, New Taipei City 243, Taiwan
[4] Mechanical Engineering Department, Faculty of Engineering and Quantity Surveying, INTI International University, Nilai 71800, Negeri Sembilan, Malaysia
[5] College of Engineering and Center for Green Technology, Chang Gung University, Taoyuan 333, Taiwan
* Correspondence: cpchen@mail.mcut.edu.tw (C.-P.C.); chensc@mail.mcut.edu.tw (S.-C.C.)

Experimental Details

Device Fabrication

The ITO substrate was manufactured by Sanyo, Osaka, Japan (8 Ω/□) and cleaned before deposition by sonication in soap, deionized (DI) water solution, acetone, and isopropyl alcohol (IPA) for 30 min. The $MAPbI_3$ (perovskite) precursor solution was prepared by dissolving 1.3 M PbI_2 and MAI (molar ratio, 1:1) in anhydrous DMF/DMSO (4:1). The incorporation of urea in a perovskite precursor solution can promote the growth of perovskite grains and passivate the defects at the grain boundaries and, thereby, enhance the PV performance [R1-R3]. The solutions were then spin-coated on the Cu_2O-coated substrates with a two-step spin rate (step 1: 2000 rpm for 10 s; step 2: 4000 rpm for 25 s), and then toluene (300 μL) was used as antisolvent to induce fast crystallization. Finally, the sample was annealed at 130 °C for 10 min to complete the transformation to the perovskite. $PC_{61}BM$ (20 mg mL^{-1} in anhydrous chlorobenzene) was deposited; following the deposition of BCP (2 mg mL^{-1} in IPA), spin-coating was performed at 6000 rpm for 10 s. Finally, the device was completed through the evaporation of Ag contact electrodes (100 nm) at a vacuum level of 10^{-7} Pa through a shadow mask. The active area of this electrode is fixed at 10 mm^2.

Characterization

The thickness of the deposited Cu_2O films was monitored by α-step (ET-200). Current-voltage (I–V) properties of the devices were measured inside a glove box using a computer-controlled Keithley 2400 source measurement unit (SMU) and an Enlitech simulator (AAA Class Solar Simulators) under AM 1.5G illumination (1000 Wm^{-2}) used to determine PCEs. For simulated indoor measurements, the performance was determined under a TL84 fluorescent light (1000 lux illumination). The input power of the 1000 lux fluorescent lamps was determined using both a Lux meter (TES-1332A) and a StellarNet BLUE-Wave Spectrometer. Before measuring the light intensity at 1000 lux, the signal in the dark was recorded as the background. The dark spectrum was used to compensate for the instrument noise and dark current thermal drift. The light intensity at 1,000 lux was then calibrated with the dark signal. For indoor lighting, the Lux meter and spectrometer used the following standards as calibration reference standards: CIE S063/CIE S023/ISO 19476/SEMI PV80. An X-ray diffractometer (XRD, Malvern PANalytical Empyrean, Malvern) with Grazing Incidence X-ray Diffraction (GIXRD) using the Cu Kα radiation (the wavelength is 1.5406 Å) was used for the identification of the phase structure. Films' surface roughness was measured by an atomic force microscope (AFM, Bruker Dimension Edge). Films' compositions were analyzed using a JEOL JXA-8200 (Tokyo, Japan) electron probe X-ray microanalyzer (EPMA). The work functions were calculated using an incident light energy of 21.2 eV [He(I) emission] by UPS (Thermo VG Scientific Company). The samples were biased at –5 V dc to drive low-energy secondary electrons into the detector.

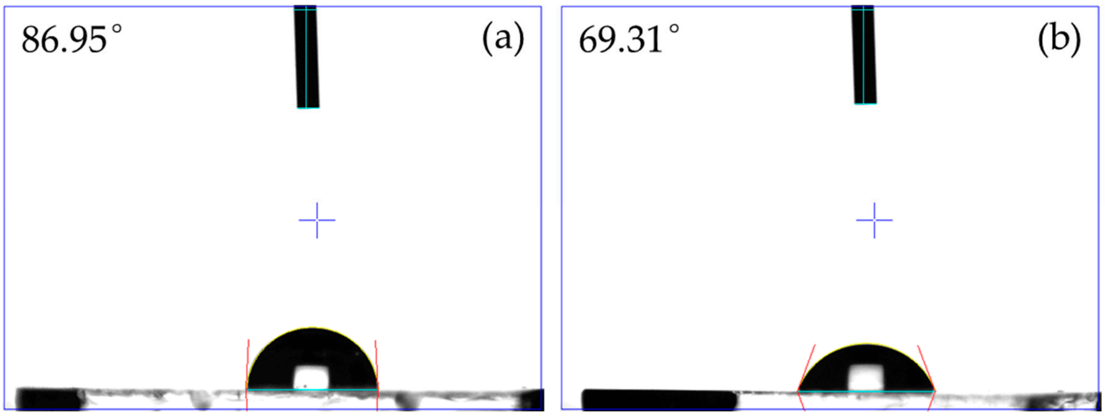

Figure S1. Cu_2O film prepared by (a) DCMS, (b) superimposed HiPIMS of contact angle.

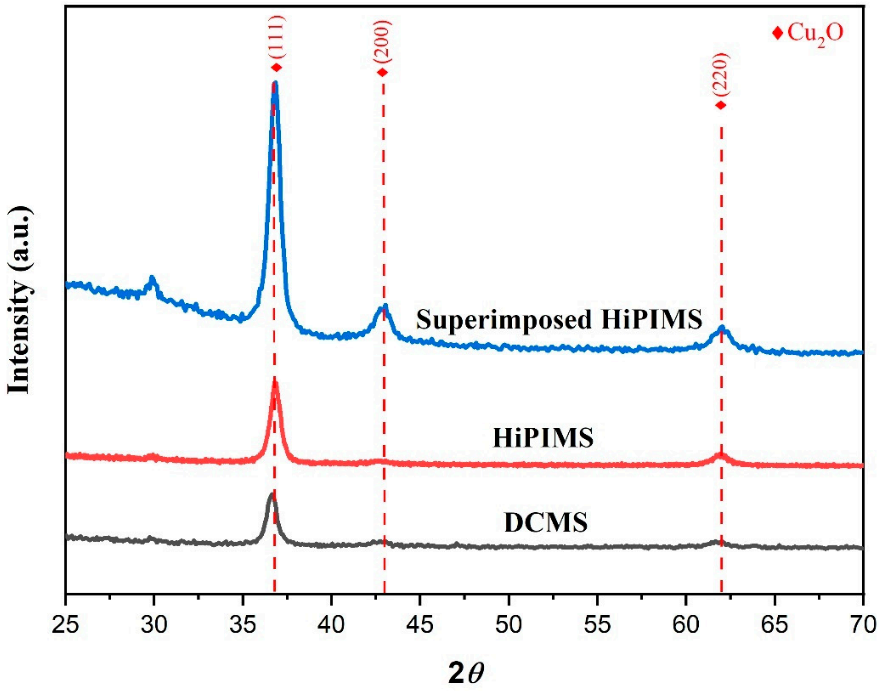

Figure S2. X-ray diffraction spectra of different process technologies.

Table S1. Chemical composition of different process technologies.

Samples	Cu (at.%)	O (at.%)	Cu/O ratio
DCMS	71.7	28.3	2.5
HiPIMS	70.2	29.8	2.4
Superimposed HiPIMS	65.4	34.6	1.9

Reference

[R1] H.L. Hsu; H.T. Hsiao; T.Y. Juang; B.H. Jiang; S.C. Chen; R.J. Jeng; C.P. Chen. Carbon nanodot additives realize high-performance air-stable p–i–n perovskite solar cells providing efficiencies of up to 20.2%, *Adv. Energy Mater.* **2018**, 8, 1802323.
[R2] J.W. Lee; S.H. Bae; Y.T. Hsieh; N. De Marco; M. Wang; P. Sun; Y. Yang. A bifunctional lewis base additive for microscopic homogeneity in perovskite solar cells, *Chem*. **2017**, 3, 290-302.
[R3] C.M. Hsieh; Y.S. Liao; Y.R. Lin; C.P. Chen; C.M. Tsai; E.W.G. Diau; S.C. Chuang. Low-temperature, simple and efficient preparation of perovskite solar cells using Lewis bases urea and thiourea as additives: stimulating large grain growth and providing a PCE up to 18.8%, *RSC Adv.* **2018**, 8, 19610-19615.
